# Indents

**Published:** 1903-06-15

**Authors:** 


### INDENTS.

☞ **Brief Articles of Technical or Scientific Value will receive notice and comment. Contributions invited.**

**Neuralgia Lotion.**

℞ Oil of Peppermint............8 parts.
Tincture of Aconite..........4 parts.
Chloroform....................2 parts.

Apply to skin only.

—*Pacific Drug Review.*

**Adrenalin as a Resuscitant.** The remarkable effect of adrenalin upon the heart, observed clinically as well as experimentally, suggests its use in conjunction with artificial respiration in the treatment of asphyxia neonatorum. The 1:1000 solution is said to be promptly absorbed from the mouth.—*Therapeutic Notes.*

**Neuralgia.** Compressed tablet No. 394, Analgesine, Parke, Davis & Co., has the following formula:

Acetanilid, 3 grains.

Ammonium Chloride, 1 grain.

Citrated Caffeine, ½ grain.

Sodium Bicarbonate, ½ grain.

—*Therapeutic Notes.*

**A New Obtundent.** Dr. Hofheinz, of Rochester, N. Y., suggests the use of zinc chloride in an alcoholic and chloroform solution instead of an aqueous one, for the treatment of hypersensitive dentine. The pain is greatly lessened, and it acts more rapidly owing to the dessicating and obtunding effect of the chloroform and alcohol.—*Dental Cosmos*, January, 1903, page 33.

**To Clean Molten Zinc.** On page 508 of the April issue I notice a suggestion to overcome the deterioration in molten zinc. My method of treating dirty zinc is as follows: When melted, add a teaspoonful or two of muriate of ammonia, stir briskly with a clean iron spoon, skim off the dirt, and the zinc will pour like water and at a lower temperature than before it was cleansed. The same process will purify tin, lead or Babbit metal.—B. E. Mead, Greenwich, Conn., in *Digest*.

**Somnoform.** The author has anesthetized forty patients with this anesthetic. The time required to bring about anesthesia varies from thirty to fifty seconds; the color of the face remains normal; the pulse is quick at first, but rapidly becomes regular; no excitement takes place except some contractions of the hands. The patient regains consciousness easily and without experiencing any malaise. The author expresses a favorable opinion of this agent, especially when contrasted with ethyl bromid.—Dr. Pitcsh, *Revue de Stomatologie*.

**The Value of a Tooth.** Why a person should be as reluctant to lose a tooth as to lose a finger.

"What's a tooth, anyway? If it hurts, yank'er out."

What's a hobnail in your boot? If it gets sharp, bent and catching into things, yank'er out.

One is about as important as the other—with some folks.

Say, I know a dentist who, long years ago, early in his practice, took out thirty-two teeth from one mouth in one sitting. Wow!

He did it under protest, but was sorry he didn't positively refuse before he was fairly begun.—R. B. Tuller, in *American Journal*.

**An Oxyacetylene Blow=Pipe.** An oxyacetylene blow-pipe is described by M. Fouche in the Bulletin of the French Physical Society. The flame is formed by the combustion of a mixture of one part of acetylene to one-eighth of oxygen, and in order that the explosion may not travel back into the blow-pipe, a jet velocity is required due to the pressure of a water column four metres in height. The flame melts most metals readily; it will solder iron and steel. Even silica lime are melted by it. With a reduction of the proportions of oxygen, the flame becomes luminous, and on falling on lime the free carbon goes to form carbide of lime. —*Scientific American.*

**Obtunding Dentin.** Continuously we hear about new local obtundents for the painless excavation of hypersensitive dentine. Here is a combination of drugs that is second to none: Melt together in a test-tube equal parts of menthol and cocain hydrochlorate and add to it an equal amount of liquid carbolic acid. Keep in well stoppered colored bottles. Before applying the heated solution to the dentin the cavity should be washed with a warmed alkaline solution, to neutralize any acids present, and dried with alcohol and the hot-air blast. This mixture is also of marked benefit in reducing the pain in fitting bands and in removing deep-seated calcareous deposits from roots in the treatment of pyorrhea.—Editorial in *Dental Era.*

**Refitting a Lower Vulcanite Plate.** Gum sections having been used, and not wishing to disturb the well-made joints, I removed with file and saw the entire lower portion of the plate. It was then roughened so that impression material would readily adhere to it and a sufficient amount prepared and placed upon it. In this condition the plates were placed in the mouth and the lady asked to close her jaws upon them. The compound was then cooled as quickly as possible and the plate removed. After thoroughly chilling the impression material it was trimmed and finished as if it were a completed case. Thoroughly chilling it again it was placed in the mouth and the lady given some crackers to eat. Everything seemed to be working satisfactorily and the case was then flasked and a new bottom vulcanized upon it. This, up to the present, has been very satisfactory and the method may be taken and used upon its merits.—J. M. Thompson, in *American Journal.*

**Blackmail.** A woman in Waterloo, Iowa, has sued a prominent dentist of the town for $3,000 damages, alleging criminal assault while she was under the influence of an anesthetic.—A dentist at Rockaway Beach, N. Y., has been accused by a young girl of the same crime.—A woman at Leadville, Colo., has sued a dentist on the same charge. In all three cases the dentists charge blackmail, and circumstances point to their innocence. We would again repeat what we have frequently urged in the past, namely, that a dentist should never administer an anesthetic to a woman without a third party being present. Usually these suits are blackmail pure and simple, but occasionally a respectable woman will come out from under the anesthetic with some hallucination, and the dent'st's only safeguard in any event is the testimony of a third person.—*Digest.*

**Adrenalin as a Hemostatic.** With 25 Cc, of solution adrenalin chloride the author was able to save the lives of six patients who were threatened with death from hemorrhage. The first patient was a hemophilic young man, constantly bleeding from a cut in the thumb. The hemorrhage was checked for twenty-four hours, by the use of gelatine internally and by the rectum, but recurred. The wound was the dressed with gauze impregnated with 1:2000 adrenalin solution, when the hemorrhage stopped at once.

The same result followed the application of the gauze in two cases of hemorrhage during the changing of the dressings of an appendicitis incision. The secretion from the wound was much decreased after the application of adrenalin. Two other patients received adrenalin internally in the treatment of recurring hemoptysis or hematemesis. After taking 30 drops of adrenalin twice in two hours, the hemoptysis ceased permanently. The same effect followed in a case of morning hematemesis from a gastric cancer.—Dr. Lange, in *Therapeutic Notes.*

**Damage Suits.** A man at Salem, Mass., has sued a hospital in that town for $10,000 damages, alleging that whiie a patient in the institution ether was carelessly administered in an operation for appendicitis, and because of it he swallowed his false teeth.—A young woman is sueing Dr. J. R. Burdick, a dentist at Omaha, for $50,000 damages for breach of promise.—Through his guardian, a boy at Los Angeles, Cal., has brought suit for $5,000 damages against a dental parlor, alleging permanent injuries from unskillful work.—A verdict of $125 has been awarded against a dentist of Providence, R. I. The plaintiff alleged incompetency.—A woman at Worcester, Mass., has sued a dental parlor for $1,000 damages, alleging that the proprietor broke her jaw in extracting a tooth.—A woman in Seattle, Wash., has sued a dentist for $1,000 damages for injuries due to careless work.—A man at Providence, R. I., has sued his dentist for $2,000 damages, alleging injuries.—A woman at Syracuse, N. Y., was awarded $50 damages against a dentist, whom she alleged, had pulled a sound tooth —*Digest.*

**Adhesive Aids in Starting Gold Filling.** Lately this method has been revived by the placing upon the market of two preparations intended to take the place of the mechanical expedients for the starting of fillings.

One of these, known as Kerr's Sealer, is a solution of pure caoutchouc in a menstruum which readily evaporates; while the other, designated auro-percha, consists of small granules of gutta-percha.

The Sealer is best applied by wrapping a minute portion of cotton around the point of a small broach or canal plugger, dipping this into the solution and then touching the spot in the cavity selected for the beginning of the filling.

Before the volatile portion has time to evaporate a piece of gold (crystal gold seems to be the most suitable) is placed upon it and partially condensed. In a few moments perfect adhesion will have been secured between the gold and cavity wall, permitting the filling to be proceeded with in the usual manner.

In using the auro-percha the first piece of gold is warmed over the flame of an annealing lamp, then touched to a few granules of the material which will adhere to it, warmed again to thoroughly soften the gutta-percha and quickly pressed to place in the cavity and condensed. Sufficient hardening of the substance to secure it in position soon takes place when the filling can be carried to completion.—S. H. Guilford, in *Stomatologist.*

**Duplicating a Vulcanite Denture.** To make a new piece which shall be just like the one already made, plus the alteration which has made remaking necessary, proceed as follows: Having made good the defective part with wax, tooth, etc., pour plaster into the piece and put it into the lower half of a flask (teeth upward.) Then smooth plaster, and when set, soap or oil it. Now build plaster all around the gum (if any) and teeth to the tops of the teeth. When the plaster is set, shape it, slanting from the tops of the teeth down to the edge of the flask, and cut three V-shaped notches, one opposite the central incisors and one opposite each pair of bicuspids, cutting almost through to the teeth. Then soap and fill up flask, not shaking the plaster down, but using thin plaster and letting it flow down. When the plaster is set the flask is opened and a few smart blows struck on the sides with a mallet. This causes the rim of plaster surrounding the teeth to fracture at the notches. The four pieces are placed in position in the upper half of the flask, the teeth are taken out of the vulcanite, put in place, and the case packed in the usual way.

When finished the piece will be as nearly as possible what the other was intended to be. An excellent way of taking teeth out of vulcanite is as follows: Fill a ladle with dry plaster, bury the piece in it, put on the fire or stove. When hot, hold the piece in tongs or pliers and push off the teeth.—D. Laville, in *Journal British Dental Association.*

